# A Novel Breakthrough in *Leptospira* spp. Mutagenesis: Knockout by Combination of CRISPR/Cas9 and Non-homologous End-Joining Systems

**DOI:** 10.3389/fmicb.2022.915382

**Published:** 2022-05-26

**Authors:** Luis G. V. Fernandes, Ana L. T. O. Nascimento

**Affiliations:** ^1^Laboratorio de Desenvolvimento de Vacinas, Instituto Butantan, São Paulo, Brazil; ^2^Programa de Pos-Graduacao Interunidades em Biotecnologia, Instituto de Ciencias Biomedicas, São Paulo, Brazil

**Keywords:** *Leptospira interrogans*, leptospirosis, CRISPR/Cas9, *Mycobacterium tuberculosis*, *Mycobacterium smegmatis*, non-homologous end-joining, knockout mutants, *Leptospira biflexa*

## Abstract

Leptospirosis is of general concern as it is a widespread zoonotic disease caused by pathogenic species of the genus *Leptospira*, although this genus also includes free-living saprophytic strains. Understanding the pathophysiology of leptospirosis is still in its infancy even after several years of its discovery, because of the lack of effective genetic tools. The use of the *Streptococcus pyogenes* CRISPR/Cas9 system and its variations have pushed the leptospirosis research forward, relying on the simplicity of the technique. However, the lethality of double-strand breaks (DSB) induced by the RNA-guided Cas9 enzyme has limited the generation of knockout mutants. In this work, we demonstrated sustained cell viability after concurrent expression of CRISPR/Cas9 and *Mycobacterium tuberculosis* non-homologous end-joining components in a single-plasmid strategy in *L. biflexa*. Scarless mutations resulting in null phenotypes could be observed in most of the colonies recovered, with deletions in the junctional site ranging from 3 to almost 400 bp. After plasmid curing by *in vitro* passages in a medium without antibiotic, selected marker-free and targeted mutants could be recovered. Knockout mutants for LipL32 protein in the pathogen *L. interrogans* could be obtained using *M. smegmatis* NHEJ machinery, with deletions ranging from 10 to 345 bp. In conclusion, we now have a powerful genetic tool for generating scarless and markerless knockout mutants for both saprophytic and pathogenic strains of *Leptospira*.

## Introduction

Leptospirosis is a zoonotic disease of global impact and is caused by the pathogenic species of the genus *Leptospira*, which also includes free-living saprophytic strains (Levett, 2001; Bharti et al., 2003; Haake and Levett, 2015). Rodents are chronic reservoirs of pathogenic bacteria and therefore the main carriers of the disease, due to colonization in the kidneys. Infection occurs *via* contact with the urine of infected hosts, either directly or indirectly through contaminated water or soil (Ko et al., 2009). Humans and other animals can be infected through intact sodden or damaged skin or mucosa, exhibiting from a wide range of non-specific symptoms, such as fever, chills, headache, and myalgia, to severe leptospirosis, a potentially fatal condition known as Weil’s disease, corresponding to 5–15% of the reported cases (Bharti et al., 2003; Costa et al., 2015). Leptospirosis is responsible for more than one million cases and 60,000 deaths per year worldwide (Costa et al., 2015).

Elucidation of leptospirosis pathogenesis and subjacent virulence factors is still considered incipient, with only a few avirulent mutants described to date (Ristow et al., 2007; Murray et al., 2009b; Adler et al., 2011; Eshghi et al., 2012; Lambert et al., 2012; Pappas and Picardeau, 2015). The generation of specific, targeted mutations and evaluation of resulting phenotypes are key steps to a better and comprehensive understanding of disease progression and identification of leptospiral proteins involved in the infection process, ultimately paving the way to the development of better therapeutic and prophylactic strategies.

Significant progress was made in the past years regarding genetic manipulation of *Leptospira* species, favored by the generation of a *Leptospira-E. coli* shuttle vector, pMaOri (Pappas et al., 2015); this plasmid allowed the episomal delivery of the *Streptococcus pyogenes* type II CRISPR/Cas (clustered regularly interspaced short palindromic repeat/CRISPR associated) system. The two-component simplicity, exemplified by the requirement of only reprogrammable DNA endonuclease activity of Cas9 nuclease for DNA cleavage and an engineered single-guide RNA (sgRNA) for target specificity, has emphasized the applicability of this system as a biotechnological tool for genome engineering (Jinek et al., 2012; Jiang et al., 2013, 2015).

The recognition of a 3′ protospacer adjacent motif (PAM) by Cas9 and subsequent RNA–DNA Watson and Crick base pairing led to DNA cleavage, provoking double-strand breaks (DSBs) that need to be repaired for cell viability (Shuman and Glickman, 2007; Lieber, 2010). Most bacteria are unable to resolve DSB in the absence of a template for recombination, and Cas9 cleavage has been previously demonstrated to be lethal to *Leptospira* (Fernandes et al., 2019).

The first strategy applied to overcome this lethality in both saprophytic (Fernandes et al., 2019) and pathogenic (Fernandes et al., 2022) strains was the expression of a catalytically dead Cas9 (dCas9) and a sgRNA capable of pairing to the coding strand of the desired gene, as the dCas9–sgRNA complex functions as a physical barrier, interfering with RNA polymerase elongation and resulting in gene silencing rather than disruption (Qi et al., 2013; Choudhary et al., 2015; Zhao et al., 2017). Generally, there are two distinct pathways to repair induced DSBs: non-homologous end-joining (NHEJ) and homology-directed repair (HDR) (Choulika et al., 1995; Weterings and van Gent, 2004; Lieber, 2010). Despite HDR being an advantageous strategy for inducing specific point mutations, the low availability of replicative plasmids for pathogenic *Leptospira* and validated antibiotic resistance markers (Poggi et al., 2010) has pushed the research interests away from this strategy.

In eukaryotes, the NHEJ system is the leading DNA repair system to sustain genome stability (Mao et al., 2008), directly ligating broken DNA ends, as this pathway displays no sequence requirements (Bowater and Doherty, 2006). Since the NHEJ is an error-prone mechanism (Kim and Kim, 2014), insertion and/or deletion (indel) mutations are expected to occur at the junctional site after DNA ligation (Bassing and Alt, 2004), thus inducing the formation of a premature stop codon, which is desirable when null phenotypes are pursued, and this is a more straightforward strategy than HDR (Choi and Lee, 2016). Contrarily to the complexity of the eukaryotic NHEJ system, some bacteria (*M. tuberculosis*, *M. smegmatis, Pseudomonas aeruginosa, Bacillus anthracis*, and *B. subtilis*) express a simpler version of this pathway, which is composed of two key proteins: an ATP-dependent ligase (LigD) and a DNA end-binding protein Ku (Shuman and Glickman, 2007; Aniukwu et al., 2008; Matthews and Simmons, 2014). In this sense, the NHEJ system of *M. tuberculosis* and *M. smegmatis* has been shown to be functional in some bacteria (Malyarchuk et al., 2007; Tong et al., 2015; Su et al., 2016; Zheng et al., 2017). So far, the applicability of the heterologously expressed NHEJ system in *Leptospira* spp. has not yet been explored.

In this work, we demonstrated that DSB lethality in *L*. *biflexa* could be surpassed by the concomitant expression of *S. pyogenes* CRISPR/Cas9 and *M. tuberculosis* NHEJ systems, with null phenotypes being observed in most recovered colonies, which is compatible to the indel mutations observed in the selected genes. Plasmid elimination by *in vitro* passage resulted in the recovery of scarless, resistance marker-free, targeted mutants. Application of this strategy to the pathogen *L. interrogans* required the use of *M. smegmatis* NHEJ system, where distinct clones of LipL32 protein knockout mutants were obtained. This strategy is used for the first time in *Leptospira*, opening new windows of opportunity for studying these bacteria.

## Materials and Methods

### Bacterial Strains and Media

Saprophytic *L. biflexa* serovar Patoc strain Patoc1 and low-passage pathogen *L. interrogans* serovar Copenhageni strain Fiocruz L1-130 were cultured in EMJH medium (Difco, BD, Franklin Lakes, NJ, United States) supplemented with 10% (vol/vol) *Leptospira* Enrichment EMJH (Difco) (Turner, 1970). Solid media were prepared by supplementing with 1.2% noble agar (Difco). Where necessary, spectinomycin was added at a concentration of 40 μg/ml. *E. coli* auxotrophic strains π1 and β2163 (Demarre et al., 2005; Picardeau, 2008) were used for general cloning and as conjugation donor cells, respectively, and were grown in Luria–Bertani (LB, Difco) medium. For *E. coli* strain π1, thymidine (dT, 0.3 mM, Sigma) was added to the medium, and for strain β2163, diaminopimelic acid (DAP, 0.3 mM, Sigma) was added.

### Construction of Plasmids Containing CRISPR/Cas9 and NHEJ Systems

**FIGURE 1:**
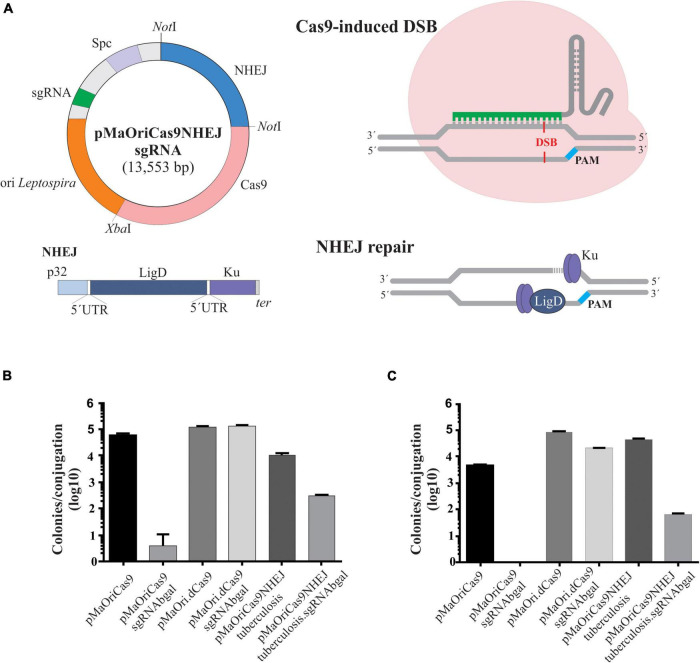
Description of NHEJ repair strategy and assessment of transconjugant recovery in *L. biflexa*. **(A)** NHEJ cassette, comprising *M. tuberculosis ligD* and *ku* genes under the control of leptospiral *lipL32* promoter, was ligated into pMaOriCas9 plasmids. Programmable Cas9 enzyme, guided by the sgRNA, will provoke a DSB in the desired genomic target, which would be further repaired by the action of LigD and Ku proteins. **(B,C)** Distinct plasmids were transferred to *L. biflexa* cells by conjugation with *E. coli* β2163 and then plated onto EMJH plus spectinomycin plates at different dilutions for cell enumeration. Values are expressed in colonies per conjugation ion log scale.

Coding sequences of the *ligD* and *ku* genes of the NHEJ system from *M. tuberculosis* and *M. smegmatis* were obtained from GenBank^1^. The expression cassette was composed of the constitutive and strong *lipL32* promoter (p32) [334 nucleotides upstream of the TSS, previously defined by Zhukova et al. (2017)], 5′ UTR from the same gene, including the putative ribosome binding site (RBS), *ligD* coding region, repetition of the 5′UTR, and then *ku* coding sequence, followed by *Borrelia burgdorferi bmpB* transcription terminator (Jewett et al., 2011; Figure 1A). Both coding sequences were codon-optimized for *Leptospira* spp., and the final cassette was synthetized by GeneArt (Invitrogen), with flanking *Not*I restriction sites. The synthetic gene and the plasmid pMaOriCas9 (Fernandes et al., 2019) were digested with *Not*I enzyme for 2 h at 37°C, and fragments were purified from the reaction by using GFX™ PCR DNA and Gel Band Purification Kit (GE). The plasmid and insert were then mixed at a 1:10 proportion for T4 ligase-mediated ligation (Thermo). Reaction mixtures were used to transform *E. coli* π1 cells, which were seeded onto LB medium plus spectinomycin (40 μg/ml) and dT (0.3 mM). Colonies were randomly selected, grown in a liquid medium with the same supplementation for plasmid extraction with the illustra plasmidPrep Mini Spin Kit (GE), and recombinant plasmids were confirmed by restriction digestion with *Not*I. The final plasmids were designated pMaOriCas9NHEJtuberculosis and NHEJsmegmatis and further used for ligation of the sgRNA cassette targeting leptospiral β-galactosidase (LEPBI_RS00135) or pathogen-specific *lipL32* (LIC_RS06940) at the *Xma*I site, according to Fernandes et al. (2019). β-galactosidase sgRNA cassette contains the 20-nt sequence 5′ AATTTGCCAACCTACAACCG 3′, and *lipL32* sgRNA contains the 20-nt sequence 5′ ACCACCGAAAGCACCACAAG 3′ as protospacer. Plasmids pMaOriCas9 and pMaOri.dCas9 (Fernandes et al., 2019), alone or containing the same sgRNA cassettes, were also employed as controls in the transformation experiments.

### *Leptospira*–*E. coli* Conjugation

The conjugative *E. coli* strain β2163 was used to deliver the plasmids to *Leptospira* spp. by conjugation, as previously described (Picardeau, 2008; Fernandes et al., 2022). Saturated *E. coli* cultures containing each of the desired plasmids were diluted 1:100 in fresh LB medium supplemented with DAP, until an optical density of 0.2–0.4 at 420 nm was obtained, and then mixed at 1:1 cell proportion with either *L. biflexa* or *L. interrogans*, which were used when cultures reached OD_420nm_ of 0.2–0.4 in EMJH medium, corresponding to approximately 2–5 × 10^8^ cells/ml. Cells were concentrated on a 25-mm diameter, 0.1-μm pore size mixed cellulose esters membrane (Millipore, VCWP02500) using a filtration apparatus connected to a vacuum pump. Membranes were recovered and incubated on EMJH plates supplemented with DAP for 24 h at 30°C, bacterial side up, and cells were then recovered from the filters by placing them into 50-ml conical tubes and vigorous pipetting with 1 ml EMJH. Different volumes of the mixed cultures (10, 100, or 400 μl) were spread on EMJH plates containing spectinomycin for the selection of recombinant leptospires. After incubation at 30°C for 10 days, colonies were retrieved from plates, vortexed in 100 μl of EMJH for microscopic confirmation of live and motile cells, and finally inoculated in liquid EMJH plus spectinomycin. Mid-log phase cultures were then validated for the presence of the plasmids by PCR with pMaOri2 primers (Table 1) flanking the sgRNA insertion site.

**TABLE 1:** Primer sequences used in this work.

Primer	Sequence (5′→3′)	Context of use
pMaOri2 F	ACGCAATGTATCGATACCGAC	Amplification flanking the sgRNA cassette
pMaOri2 R	ATAGGTGAAGTAGGCCCACCC	
aadA F	CGATTCAAACATTAAAAATCG	qPCR targeting the spectinomycin resistance gene
aadA R	CACTCACCGTATATAAATTCTC	
dnaK F	GATTTGGGAACTGGCAAAGA	qPCR targeting the *dnaK* gene
dnaK R	TGGCTTCCAATTCGTTTTTC	
pMaOri F	AGTGACACAGGAACACTTAACG	Demonstration of NHEJ cassette ligation into pMaOriCas9
NHEJ R	GTACCATGTGTTGCTAAC	
β-galactosidase F1	CTTTGGAGCCTGTTATTACC	Amplification of β-galactosidase gene in *L. biflexa*
β-galactosidase R1	TGAAAACTTCAGTCCATCAC	
β-galactosidase F2	CTCTCTTCGATGCAATCTTAG	
β-galactosidase R2	ACTCATCAGATTGGAAACG	
LipL32 F1	GTCAGAGAATTATTGAAGAG	Amplification of *lipL32* gene in *L. interrogans*
LipL32 R1	GAACTGGTTTTGCTTTCGC	
LipL32 F2	ATTATTCAATATTGATTTAC	
LipL32 R2	GCTTGTCCTGGCTTTACG	

### Screening of β-Galactosidase Null Mutants and Validation in *L. biflexa*

Colonies were randomly selected from plates containing pMaOriCas9NHEJsgRNAbgal, grown in liquid EMJH plus spectinomycin (40 μg/ml) until the exponential phase, and then harvested by centrifugation (10,000 × *g*, 15 min). Bacterial pellets, totaling 5 × 10^8^ cells, were resuspended in PBS containing the β-galactosidase chromogenic substrate X-gal (400 μg/ml) and incubated at 37°C for 6 h for the qualitative assessment of mutations. For calculating the global frequency of null mutations, sterile X-gal solution was carefully spread on the surface of plates after full colony formation and incubated for 16 h for blue/white screening.

The β-galactosidase activity was also evaluated by using o-nitrophenyl-β-D-galactopyranoside (ONPG) substrate, as previously performed by Fernandes et al. (2019). Briefly, 5 × 10^8^ cells were centrifuged, and the supernatant was discarded and cells resuspended in 1 ml of Z-buffer (pH 7, containing 130 mM monosodium phosphate, 40 mM disodium phosphate, 10 mM potassium chloride, 2 mM magnesium sulfate, and 40 mM β-mercaptoethanol). Next, 40 μl of chloroform and 30 μl of 0.1% SDS were added to the samples, which were then vigorously vortexed for 15 s and incubated at 30°C for 5 min. Finally, 200 μl of 13.27 mM ONPG (Sigma) in Z-buffer was added, and the samples were incubated at 37°C for 4 h for color development. Reactions were stopped by mixing 500 μl of the sample with 250 μl of 1 M sodium carbonate, and color change was evaluated using a spectrophotometer to measure the absorbance at 420 nm.

### Culture Passages

Confirmation of permanent mutation, in contrast to CRISPRi episomal silencing, was performed by serial passage of mutant cultures in liquid EMJH with no antibiotic selection, with an initial inoculum of 10^5^ cells/ml. At each passage, we also included mutant cultures grown in the presence of spectinomycin, or leptospires containing empty pMaOriCas9NHEJ plasmids, as a positive control for target protein expression. Cultures containing pMaOri.dCas9 with distinct sgRNA were included to show the gradual loss of silencing. For β-galactosidase mutation in *L. biflexa*, enzyme activity was revealed by ONPG substrate from two biological replicates of each group, as mentioned earlier, and the values displayed by the control containing empty plasmid were considered to indicate 100% activity at each passage.

### Quantitative PCR for Plasmid Frequency

Knockout and knockdown (episomal CRISPRi, employed as control) mutants were evaluated for plasmid upkeep in cultures grown without antibiotic pressure, using real-time quantitative PCR (qPCR) targeting the pMaOri backbone sequence. Total DNA was extracted when cultures reached the mid- to late-log phase using the DNeasy Blood and Tissue kit (Qiagen, MD, United States) following the manufacturer’s specifications, and the sample quality and concentration were assessed by using a BioSpectrometer (Thermofisher Scientific, CA, United States). qPCR was performed with the CFX96 Real-Time System (Bio-Rad, Hercules, CA, United States), using SYBR Green PCR Master Mix (Applied Biosystems, Foster City, CA, United States) and primers listed in Table 1. Reactions were performed in triplicate in a volume of 20 μl each containing 5 ng of DNA, 400 nM of each oligonucleotide, and 10 μl of SYBR Green PCR Master Mix (Applied Biosystems). Negative controls using all the reagents except DNA (NTC, no template control) were also included. Cycling conditions were as follows: 50°C for 2 min and 95°C for 10 min, followed by 40 cycles at 95°C for 15 s and 60°C for 40 s. The pMaOri backbone in the population was assessed by using a primer targeting the *aadA* gene (spectinomycin resistance, Table 1), and the percentage of cells containing different plasmids was determined using the 2^–ΔΔCT^ method, normalized to the amplification of the *dnaK* gene (Table 1). DNA extracted from plasmid-harboring cells grown *in vitro* in the presence of spectinomycin was employed as control (100% plasmids in population).

### Electrophoresis and Immunoblotting

Cultures of *L. interrogans* at mid- to late-log phase of growth were centrifuged (10,000 × *g*, 15 min), and the resulting pellet was washed twice with PBS. Protein samples were processed for SDS-PAGE on 12% acrylamide gels (Bio-Rad) as per the manufacturer’s guidelines. For immunoblotting, proteins were electrotransferred onto nitrocellulose membranes (Hybond ECL; GE Healthcare) by semidry transfer. Membranes were blocked with PBS containing 10% non-fat dried milk for 1 h and then incubated with indicated primary antibody diluted in the blocking buffer for 1 h at room temperature. Membranes were then washed three times with PBS 0.1% Tween 20 (PBS-T) and incubated with horseradish peroxidase-conjugated secondary anti-mouse IgG (1:5,000) antibody in a blocking buffer for 1 h at room temperature. After extensive washing, antigen reactivity was detected using SuperSignal™ West Dura Extended Duration Substrate (Thermo Scientific), and the luminescence generated was measured with the aid of an Amersham Imager 600 (GE).

### Analysis of Mutation

Distinct mutant *Leptospira* spp. cultures were used for total DNA extraction by DNeasy Blood and Tissue kit. Confirmation of genomic mutation was achieved by PCR of the desired gene using distinct combinations of primer flanking the Cas9 cleavage site (Table 1). PCR reactions were analyzed by using 1% agarose gel. Amplicons were then purified, and 100 ng of the final product was used for Sanger DNA sequencing (Sanger et al., 1977) with the same primers used for amplification. In the case of short *in-frame* mutation resulting in a null phenotype, protein modeling was used to deduce the probable subjacent cause of lack of β-galactosidase activity. Accordingly, wild-type and mutant protein sequences were submitted to the I-TASSER (Zhang, 2008) web server for 3D structure modeling, and the models with the best confidence score and Z-score were chosen and then visualized using the PyMOL (The PyMOL Molecular Graphics System, Version 2.0 Schrödinger, LLC) molecular graphics system.

### Plasmid Elimination and Screening of Selection Marker-Free Mutants in *L. biflexa*

When serial *in vitro* passages resulted in more than 50% plasmid loss in the population, as per qPCR determination, cultures were set at 10^4^ cells/ml, and 100 μl was used to seed EMJH plates with or without spectinomycin. Plates were incubated at 29°C for 10 days, and the colonies obtained when the antibiotic was omitted were randomly selected, and each of them was grown in liquid EMJH with or without spectinomycin. When growth was exclusively observed in EMJH without antibiotic, cultures were double-checked by PCR with primers pMaOri2 F and R targeting the plasmid. Subsequent to a lack of amplification, clones were again phenotypically assessed for the desired mutation, and then cultures were considered free of plasmid, thus constituting a selection marker-free, scarless knockout mutant. Mutations were re-assessed by sequencing.

## Results

### Co-expression of *M. tuberculosis* LigD and Ku Protein Allows the Recovery of Colonies After DNA Cleavage in *L. biflexa*

Cas9-induced DSB is lethal to *Leptospira* species, in accordance with the lack of orthologs to the genes involved in prokaryotic NHEJ in their genome (Nascimento et al., 2004). In contrast to the eukaryotic NHEJ system, which consists of several proteins, prokaryotic organisms exhibit a simpler NHEJ system that requires only two key proteins, LigD and Ku. Among the few bacteria that code for these proteins, the NHEJ system from *M. tuberculosis* has been previously applied for resolving DSB in *E. coli* (Su et al., 2016).

A plasmid for the concurrent expression of the *S. pyogenes* CRISPR/Cas9 and *M. tuberculosis* NHEJ systems was generated based on the pMaOriCas9 plasmid as a backbone (Fernandes et al., 2019), and hence designated as pMaOriCas9NHEJtuberculosis (Figure 1A). NHEJ genes, *ligD* and *ku*, are expressed as a bicistronic mRNA and translated separately, since an RBS was included at 5′ of each gene (Figure 1A). In this regard, DSB produced by sgRNA-driven Cas9 is expected to be repaired by the cooperative action of LigD and Ku proteins, resulting in *indel* mutation that ultimately could lead to premature stop codons.

For accessing the effect of *M. tuberculosis* NHEJ expression on the restoration of DSB, the model of saprophytic *L. biflexa* was chosen; plasmids pMaOriCas9, pMaOri.dCas9 (Fernandes et al., 2019, 2022), and newly formed pMaOriCas9NHEJtuberculosis, alone or containing the sgRNA cassette targeting the β-galactosidase gene (LEPBI_RS00135), were used for transformation. This sgRNA was previously validated in the CRISPRi system (Fernandes et al., 2019).

Two independent conjugation experiments were performed and leptospiral colonies were counted on each plate for estimating the total number of colonies per conjugation. A large number of colonies could be recovered when leptospires harbor the plasmids with no sgRNA cassette, and when it was included, a few or no colonies could be recovered in the pMaOriCas9sgRNAbgal group in two independent experiments. These results were expected based on the previous demonstration of DSB lethality (Fernandes et al., 2019) and consistent with the lack of the NHEJ system in *Leptospira* spp. (Figures 1B,C). As predicted, the expression of both dCas9 and sgRNA (pMaOri.dCas9sgRNAbgal) was tolerated by cells, since equivalent numbers of colonies could be recovered compared to the empty plasmid, and full gene silencing (knockdown) could be observed when X-gal was spread on plates (not shown).

Decreased numbers of colonies could be recovered when the pMaOriCas9NHEJ plasmid contained sgRNA in comparison to the empty plasmid (1.56 and 2.84 log reduction in the first and second experiments, respectively) (Figures 1B,C). The significant recovery of colonies when DSB was induced and the NHEJ system expressed, in contrast to the pMaOriCas9sgRNAbgal group, suggests that the DNA damage could be repaired, anticipating the success of the strategy. It is worth mentioning that when the same strategy was applied to *E. coli* by Su et al. (2016), fewer colonies were also recovered compared to when no DSB was induced (∼4 logs).

### Validation of Recovered Mutants in *L. biflexa*

Initially, four colonies from the pMaOriCas9NHEJtuberculosis.sgRNAbgal plates were randomly selected for growth in a liquid medium and subsequent validation. Groups composed of *L. biflexa* containing pMaOriCas9NHEJtuberculosis and pMaOri.dCas9sgRNAbgal (knockdown) were included as positive and negative controls for β-galactosidase activity, respectively. Co-expression of dCas9 and the sgRNA for base pairing to the coding strand of the gene by the pMaOri.dCas9sgRNAbgal plasmid results in episomal gene silencing.

**FIGURE 2:**
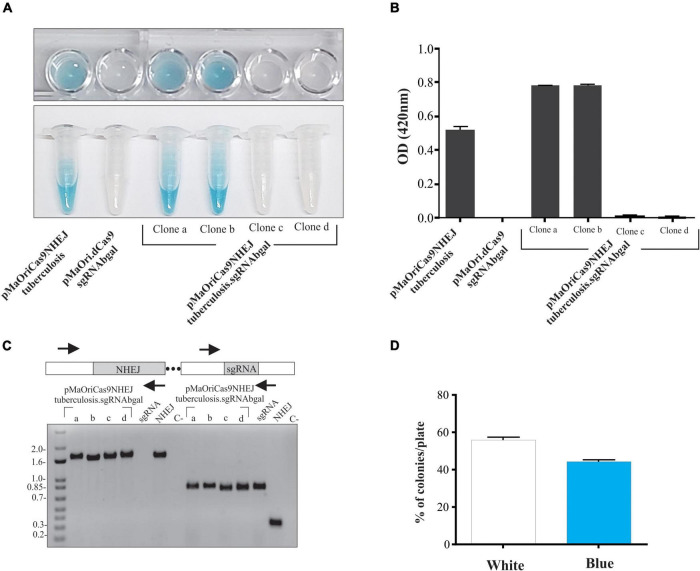
Evaluation of β-galactosidase activity in recovered recombinant *L. biflexa*. Grown cultures from individually selected colonies from pMaOriCas9NHEJtuberculosis (positive control), pMaOri.dCas9sgRNAbgal (negative control), and pMaOriCas9NHEJtuberculosis.sgRNAbgal (clones “a” to “d”) were accessed for β-galactosidase activity by X-gal **(A)** and ONPG **(B)** chromogenic substrate. Cultures were also validated by PCR with primers targeting the NHEJ and sgRNA cassette **(C)**. Plasmids pMaOri.dCas9sgRNAbgal (positive for sgRNA, no NHEJ cassette), pMaOriCas9NHEJtuberculosis (positive for NHEJ cassette, no sgRNA), and negative control (C-) with no DNA template were included. **(D)** X-gal sterile solution was spread onto plates with pMaOriCas9NHEJtuberculosis.sgRNAbgal-containing colonies for accessing the white/blue ratio.

Cultures were qualitatively evaluated by incubation with chromogenic X-gal substrate, and as expected, contrasting outcomes could be observed in the controls (Figure 2A). Two out of the four clones harvested from the plates exhibited abolished enzyme activity, strongly implying that they were knockout mutants (Figure 2A). Similar results were obtained when clones were assessed on a quantitative basis by incubation with ONPG substrate (Figure 2B). The presence of the correct plasmids within each clone was confirmed by PCR using primers targeting part of the NHEJ sequence (pMaOri F and NHEJ R) or flanking the sgRNA insertion site (pMaOri2F and R) (Figure 2C).

For calculating the percentage of mutagenesis, X-gal solution was spread on plates after colony formation for white/blue screening. The percentage of white, hereby considered knocked out colonies, was around 56% (Figure 2D), consistent with the 50% ratio observed when the colonies were randomly selected. Four additional white colonies (clones “e” to “f”) were selected for growth, and after X-gal incubation, they were confirmed to be null mutants for β-galactosidase (not shown).

### Confirmation of Permanent Phenotype by *in vitro* Passages

Despite the relatively high stability displayed by the pMaOri backbone *in vitro* (Pappas et al., 2015) and *in vivo* (Fernandes et al., 2021), it has been shown that CRISPRi episomal silencing is gradually lost after several *in vitro* passages without antibiotic selection, as a direct result of plasmid loss in the population (Fernandes et al., 2019, 2022). Thus, an experiment was designed to evaluate if the abolished β-galactosidase activity was permanent in the mutants recovered with the NHEJ system. Two different clones of each group were considered for performing the *in vitro* passages, in which media with and without spectinomycin were included. Enzyme activity displayed by pMaOriCas9NHEJtuberculosis-containing cells was considered to be 100%.

**FIGURE 3:**
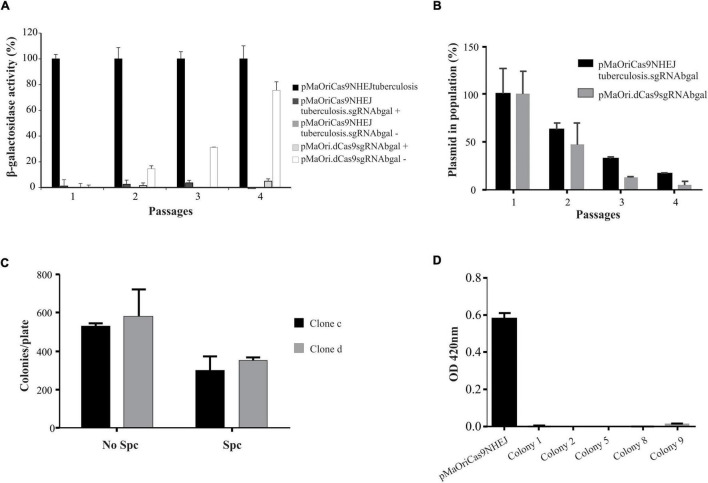
Evaluation of phenotypic stability and plasmid elimination from mutant cells. **(A)** Two clones from each group were selected for serial *in vitro* passages in media with (+) or without (–) spectinomycin. Values are expressed in average plus standard deviation (SD) from β-galactosidase activity displayed by both clones, and values from pMaOriCas9NHEJtuberculosis at each passage were considered 100%. **(B)** Cells were also evaluated by qPCR targeting the plasmid to denote its loss at each passage. After quantitative assessment of plasmid, cultures grown in the absence of antibiotic were seeded onto EMJH plates, with or without spectinomycin (Spc), counted **(C)**, and 10 colonies from clone “c” plate without Spc were randomly selected and grown in liquid media with or without Spc. Colonies that grew exclusively when Spc was omitted (colonies 1, 2, 5, 8, and 9) were revalidated for β-galactosidase activity by using ONPG substrate **(D)**.

The β-galactosidase activity started to be evident at the second passage only for the cells containing the *knockdown* system (pMaOriCas9NHEJtuberculosis.sgRNAbgal) grown without antibiotic (∼15% activity), reaching approximately 80% at passage 4 (Figure 3A), while abolished enzyme activity was consistently observed for the mutants harboring the NHEJ plasmids (Figure 3A) even when spectinomycin was omitted in the passages, supporting the permanent status of the mutations. To confirm that a stable phenotype was achieved even when plasmids were lost, cultures were assessed by qPCR targeting the pMaOri backbone (Figure 3B). As expected, leptospires harboring either pMaOri.dCas9sgRNAbgal or pMaOriCas9NHEJsgRNAbgal displayed a similar pattern of plasmid loss throughout passages in the medium without selection (Figure 3B), confirming that the permanent β-galactosidase mutation was due to the combinatory effect of Cas9 DSB and NHEJ repair.

Cultures from pMaOriCas9NHEJsgRNAbgal at passage 4 were further used for plating and screening plasmid-free colonies. Initial assessment of colony number in plates with or without spectinomycin indicated that, compatible with the qPCR results, less number of colonies were observed when plates contained antibiotic, indicating that, on average, 42% of cells in the culture were already plasmid-free (Figure 3C). Out of the 10 colonies randomly selected for growth in liquid medium from clone “c” colonies grown without selection, 5 (50%) exclusively grew when no antibiotic was added to liquid EMJH, corroborating the plasmid loss, which was confirmed by PCR (not shown), and knockout mutation was again validated by the ONPG substrate (Figure 3D).

### Assessment of Mutations in *L. biflexa* Recovered Knockout Mutants

**FIGURE 4:**
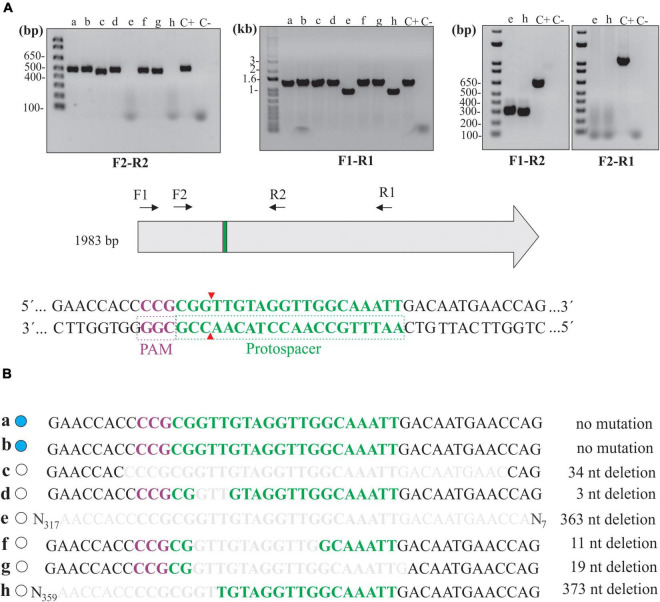
Deletion mutations at the junctional site in null mutants. **(A)** Extracted total DNA from clones “a” to “h” and from cells containing pMaOriCas9NHEJtuberculosis (C +) was amplified by PCR using a diverse array of primers, as depicted in the scheme showing the coding sequence of β-galactosidase, as well as protospacer, PAM NGG, and Cas9 cleavage site. A negative control (C-) without a template was also included. **(B)** Amplicons were sequenced with primer combinations that resulted in positive amplification and were compared to the WT sequence. β-Galactosidase status of each clone is shown on the left side (blue, WT; white, mutant) and deletion size is shown on the right.

Several knockout clones in *L. biflexa*, as per abolished β-galactosidase activity, were selected for evaluating genomic mutations within the gene following DSB repair by the NHEJ system. The combination of F2-R2 primers (Figure 4A), flanking 246 and 258 bp from the Cas9 cleavage site, respectively, resulted in a positive amplification in all the clones except for “e” and “h,” indicating that to some extent, deletion of larger fragments in these clones resulted in the loss of one or both primer annealing sites (Figure 4A). At first look, a pattern of slightly different and lower band migration indicated heterogenous deletions across samples, compared to the control in which no Cas9 cleavage occurred (pMaOriCas9NHEJtuberculosis). Other primer combinations were also used to corroborate the potential deletion of larger fragments when amplification failed with F2-R2 primers. Amplifications with F1-R1 resulted in smaller amplicons (around 350 bp) when total DNA from clones “e” and “h” was used, in line with the expected deletion of larger fragments in these cells. Next, the combined use of F1-R2 and F2-R1 primers confirmed the deletion of around 350 bp in both clones and indicated that deletions resulted in the loss of the F2 annealing site, since amplification was only observed when the F1-R2 primer set was used.

Mutations were further confirmed by sequencing of the amplicons, where assorted deletions at the repair junctions, ranging from 3 to 373 bp (Figure 4B), were observed. It is worth mentioning that no mutations were observed in clones “a” and “b,” consistent with the wild-type phenotype in these strains regarding β-galactosidase activity; however, it is unclear why no DSB occurred in these cells, since they were positive for the presence of the plasmids by PCR (Figure 2C).

Since no frameshift occurred in clone “d” (3-bp deletion, loss of a valine), protein modeling indicated that the absence of β-galactosidase activity could have been the result of subtle changes in the three-dimensional structure of the enzyme (Supplementary Figure 1). A comparison of β-galactosidase amino acid sequences between *E. coli* (1,024 aa) and *L. biflexa* (660 aa) shows that, even though they are highly divergent (5% query coverage and 37% identity), the valine in the position 134 in *L. biflexa* (the one lost in the mutation) is conserved between the two species, indicating that it may be important for enzyme activity.

In addition, after plasmid elimination in clone “c” by plating, as mentioned earlier, the mutation was re-assessed and confirmed (not shown).

### LipL32 Knockout in *L. interrogans* With *M. smegmatis* NHEJ System

Despite the successful collaborative application of CRISPR/Cas9 and *M. tuberculosis* NHEJ system in *L. biflexa* for knockout mutagenesis, significantly lower conjugation efficiency in pathogenic *L. interrogans* was anticipated to be a bottleneck in our experiments for this strain; also, it was previously demonstrated that NHEJ from *M. smegmatis* was more efficient in the recovery of *E. coli* colonies than the one from *M. tuberculosis* (Zheng et al., 2017). In this context, a new plasmid was constructed in the same manner as described for pMaOriCas9NHEJtuberculosis, in which the NHEJ system was exchanged for the *M. smegmatis* one, resulting in pMaOriCas9NHEJsmegmatis.

**FIGURE 5:**
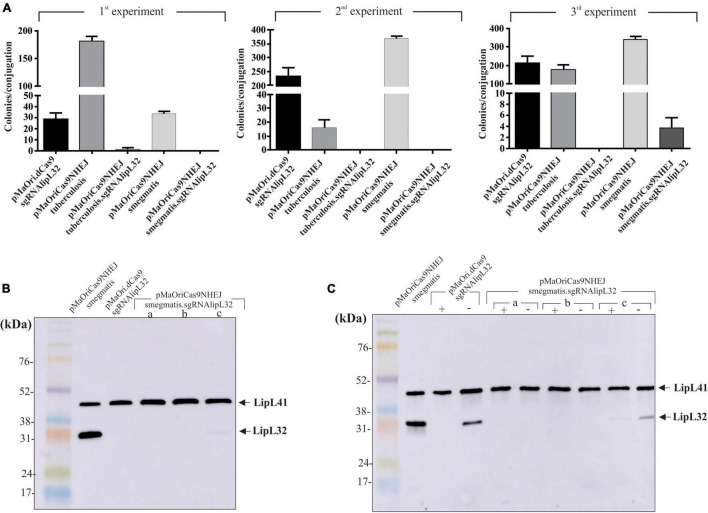
LipL32 knockout in *L. interrogans* by CRISPR/Cas9 and NHEJ from *M. smegmatis.* **(A)** Distinct plasmids were transferred to *L. interrogans* cells by conjugation with *E. coli* β2163 and then plated onto EMJH plus spectinomycin plates for cell enumeration (colonies per conjugation) in three independent experiments. Clones were recovered from plates and then evaluated by immunoblotting with anti-LipL32 (1:8,000) and anti-LipL41 (1:4,000) polyclonal antibodies for confirming LipL32 knockout. Protein profiles of clones grown in the presence of spectinomycin **(B)** or presence (+) and absence (–) of the antibiotic after five *in vitro* passages **(C)** are shown. *L. interrogans* cultures harboring pMaOriCas9NHEJsmegmatis and pMaOri.dCas9sglipL32 were included as positive and negative controls for LipL32 expression.

Plasmids alone or containing the sgRNA cassette targeting *lipL32* gene were used for *L. interrogans* conjugation; plasmid pMaOri.dCas9 containing the same sgRNA cassette was also employed as a control. As seen in Figure 5A, conjugation efficiencies were heterogeneous within the experiments and constructions, and only one colony was observed in the first experiment for the pMaOriCas9NHEJtuberculosis.sgRNAlipL32 and three colonies in the third experiment for pMaOriCas9NHEJsmegmatis.sgRNAlipL32. A higher number of colonies was observed when the NHEJ plasmids did not contain the sgRNA cassette, demonstrating that DSB is not tolerated in *L. interrogans*.

The evaluation of recovered colonies showed that the one in the *M. tuberculosis* NHEJ group was still expressing LipL32, possibly being a spontaneous mutant to spectinomycin (not shown). Coherent with the expected higher efficiency of NHEJ from *M. smegmatis*, all three colonies, when evaluated by immunoblotting, were either not expressing (clone “a” and “b”) or expressing diminished levels (clone “c”) of LipL32 protein, either when they were recovered in media with antibiotic (Figure 5B) or when they were *in vitro* cultured for five passages in medium without spectinomycin (Figure 5C), indicating the permanent status of the mutations. As expected, the *in vitro* passages of *L. interrogans* episomally silenced for LipL32 (pMaOri.dCas9sgRNAlipL32) resulted in the reappearance of LipL32 band after five passages (Figure 5C), due to plasmid loss in the population, as demonstrated for *L. biflexa* in Figure 3.

### Evaluation of *lipL32* CDS in Knockout *L. interrogans*

**FIGURE 6:**
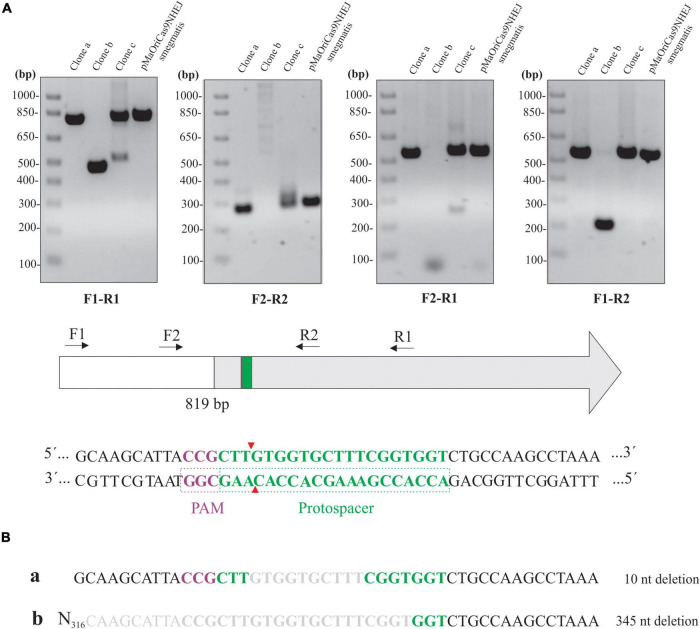
Assessment of *lipL32* sequence in *L. interrogans* mutants. **(A)** Extracted DNA from mutant clones and cells containing pMaOriCas9NHEJsmegmatis was amplified by PCR using a distinct set of primers, as depicted in the scheme showing the upstream region (white) and coding sequence of *lipL32* gene (gray), protospacer, PAM NGG, and Cas9 cleavage site. **(B)** Amplicons from F1-R1 amplification were sequenced and were compared to the WT sequence, with deletion size shown on the right.

Different primer combinations were used to demonstrate the *indel* mutation in the knockout clones for LipL32 (Figure 6A). Band patterns and no amplification at the F2-R1 primer combination in clone “b” indicate that a large deletion occurred in this clone at the 3′ site from the PAM, similar to the tendency of this directional deletion of larger fragments in *L. biflexa*. An additional band was consistently observed in clone “c” when F1-R1 and F2-R1 primers were used, and this could be the reason for the minor LipL32 signal observed in this clone by immunoblotting.

Purification and sequencing of F1-R1 amplicons showed that a 10-bp deletion occurred in clone “a,” contrasting with a 345-bp deletion in clone “b” at the repair junctions (Figure 6B). The CDS of the upstream gene to *lipL32*, LIC11353 (LIC_RS06945), was affected only in clone “b,” in which the last 42 bp of this gene were deleted; despite this polar effect, no changes in growth curves were observed (data not shown). Due to the band duplicity in clone “c,” chromatograms presented as overlapping peaks that prevented the readings.

## Discussion

Recent advances in *Leptospira* spp. mutagenesis have allowed the rapid and specific gene silencing of desired genes, supported by the type II CRISPR/Cas9 system from *S. pyogenes*. Since sgRNA-guided Cas9 genome cleavage is often lethal for prokaryotes (Citorik et al., 2014; Gomaa et al., 2014; Cui and Bikard, 2016), it was no surprise that *Leptospira* spp. could not be recovered after DSB generation (Fernandes et al., 2019), consistent with the lack of NHEJ proteins in their genomes. Lethality can be overcome by the expression of a catalytically dead Cas9 (dCas9) and sgRNA, whereas gene silencing, rather than disruption, has been achieved by hampering RNA polymerase elongation, in both saprophytic (Fernandes et al., 2019) and pathogenic strains (Fernandes et al., 2022). CRISPRi, as it stands, is an episomal, yet stable, genetic tool; however, permanent and selection marker-free mutations are still pursued.

One possible strategy to repair Cas9-induced DSB is the homology-directed repair (HDR), in which a template DNA is used to introduce specific mutations (Rudin et al., 1989; Gaj et al., 2013; Jiang et al., 2013, 2015; Tong et al., 2015). In this scenario, the need for template construction for HDR, which is time-consuming, and the limited availability of plasmids and selection markers for *Leptospira* spp. would limit the application of this repair mechanism for the recovery of knockout mutants. In addition, the low frequency of homologous recombination events in *Leptospira* would also be a bottleneck (Picardeau et al., 2001). Hence, the error-prone NHEJ system is an interesting alternative that can be exploited for inducing indel mutations for achieving knockout mutants in a single step (Shuman and Glickman, 2007; Su et al., 2016).

Only a narrow range of bacterial species, e.g., *M. tuberculosis*, *M. smegmatis*, and *P. aeruginosa*, can overcome DSB lethality by expressing the NHEJ machinery (Shuman and Glickman, 2007; Aniukwu et al., 2008; Matthews and Simmons, 2014). Prokaryotic NHEJ is composed of key functional homologs to the eukaryotic Ku70/Ku80 heterodimer and DNA ligase IV. In contrast to the eukaryotic heterodimeric Ku complex, bacterial Ku is responsible for protecting the broken DNA by forming dimers at the end of double-strand DNA (Bowater and Doherty, 2006). Here, we initially utilized the model of *L. biflexa* to validate the applicability of the heterologous and simultaneous expression of *S. pyogenes* CRISPR/Cas9 and *M. tuberculosis* NHEJ systems for obtaining knockout mutants.

When DSB was induced in *L. biflexa* cells by Cas9 cleavage, only a few colonies could be recovered, and as they exhibited the wild-type phenotype, they probably consisted of spontaneous resistant mutants, since resistance to spectinomycin in *Leptospira* has been shown to appear at the level of detection in transformation assays (Poggi et al., 2010). In contrast, concurrent heterologous expression of CRISPR/Cas9 and *M. tuberculosis* NHEJ resulted in a significant recovery of colonies, among which almost 60% displayed null phenotypes regarding β-galactosidase activity, similar to the results obtained by Su et al. (2016) by applying the same method to disrupt *lacZ* gene in *E. coli.*

Deletion mutations were observed in all mutant clones, with deleted fragments ranging from 3 to 373 bp. This discrepant deletion length was also observed by Aniukwu et al. (2008) in the native wild-type NHEJ system in *M. smegmatis* and by Su et al. (2016) when the *M. tuberculosis* NHEJ system was used to repair Cas9-induced DSB in *E. coli.* Knowing the deletion range could help the rational design of protospacers to prevent invading deletions on vicinity genes, thus avoiding off-target undesirable effects. Plasmid curing resulted in scarless, markerless, targeted mutant strains for the first time in *Leptospira* spp.

The development of knockout pathogenic strains without selection markers would be a milestone in the development of live vaccines for leptospirosis and even bacterin with the capability of Differentiating Infected from Vaccinated Individuals (DIVA). Bacterins for animal vaccines, when composed of strains lacking immunodominant proteins that are not required for virulence, as per LipL32 (Murray et al., 2009a) or LipL41 (King et al., 2013), would be ideal for differentiating infected and vaccinated animals when coupled to a diagnostic method using the lacking antigen in the knockout strain.

Targeted knockout in pathogenic *Leptospira* has been traditionally achieved by allelic exchange, using suicide plasmids to deliver a resistance marker flanked by homology “arms” to each desired target (Croda et al., 2008). In addition to the inclusion of a resistance marker to the leptospiral genome as an outcome, the requirement of double crossover events drastically reduces the frequency of knockout mutants recovered on plates, and thus, new and improved genetic techniques are pursued. In this sense, we aimed the construction of a LipL32 knockout *L. interrogans* strain in order to validate the feasibility of our current method.

Since lower conjugation efficiencies are routinely observed in pathogenic strains in comparison to saprophytic ones, we constructed another plasmid for concurrent expression of CRISPR/Cas9 and NHEJ system from *M. smegmatis*, since Zheng et al. (2017) have previously shown that its heterologous expression in *E. coli* is 45 times more efficient in rescuing cells from DSB lethality compared to the one from *M. tuberculosis*. Indeed, in our hands, mutant colonies could only be recovered when the *M. smegmatis* system was used, strengthening that NHEJ from *M. smegmatis* is more robust for repairing DSB, and the *lipL32* gene could be successfully disrupted at the vicinity of the junctional site, with deletion ranging from 10 to 345 bp. The complete characterization of these strains, including differentially expressed proteins, and their DIVA potential in animal models warrants further investigation.

In conclusion, a powerful genetic tool for selection-free knockout mutation is available for both saprophytic and pathogenic strains of *Leptospira*, and we believe that the application of this novel methodology to generate targeted mutations could aid studies in functional genomics by producing mutants for phenotype characterization and lead to the development of DIVA bacterins or even live attenuated leptospiral vaccines. Moreover, we now have a novel strategy to study and better understand the biology of *Leptospira* spp.

## Data Availability Statement

The original contributions presented in the study are included in the article/Supplementary Material, further inquiries can be directed to the corresponding author/s.

## Author Contributions

LF and AN: literature revision, manuscript preparation, and experimental design. LF: experimental manipulation and figures. Both authors reviewed and approved the manuscript, participated in the literature revision, discussion, and preparation of manuscript.

## Conflict of Interest

The authors declare that the research was conducted in the absence of any commercial or financial relationships that could be construed as a potential conflict of interest.

## Publisher’s Note

All claims expressed in this article are solely those of the authors and do not necessarily represent those of their affiliated organizations, or those of the publisher, the editors and the reviewers. Any product that may be evaluated in this article, or claim that may be made by its manufacturer, is not guaranteed or endorsed by the publisher.

## Footnotes

1
https://www.ncbi.nlm.nih.gov/genbank/

## Supplementary Material

Supplementary Figure 1 Protein modeling of wild-type and in-frame mutants of *L. biflexa* β-galactosidase. Wild-type, WT **(A)** and mutant clone “d” **(B)**, which had a 3-bp deletion resulting in the loss of a valine residue (shown in orange in the WT), are shown separately. Both structures were superimposed **(C)** and subtle changes in β-sheets could be seen close to the deletion site **(D)**.
