# Supplementary figures and images for: A Novel Breakthrough in Leptospira spp. Mutagenesis: Knockout by Combination of CRISPR/Cas9 and Non-homologous End-Joining Systems
Source: Front Microbiol. 2022 May 26;13:915382. doi: 10.3389/fmicb.2022.915382 (PMC9199861; doi:10.3389/fmicb.2022.915382)

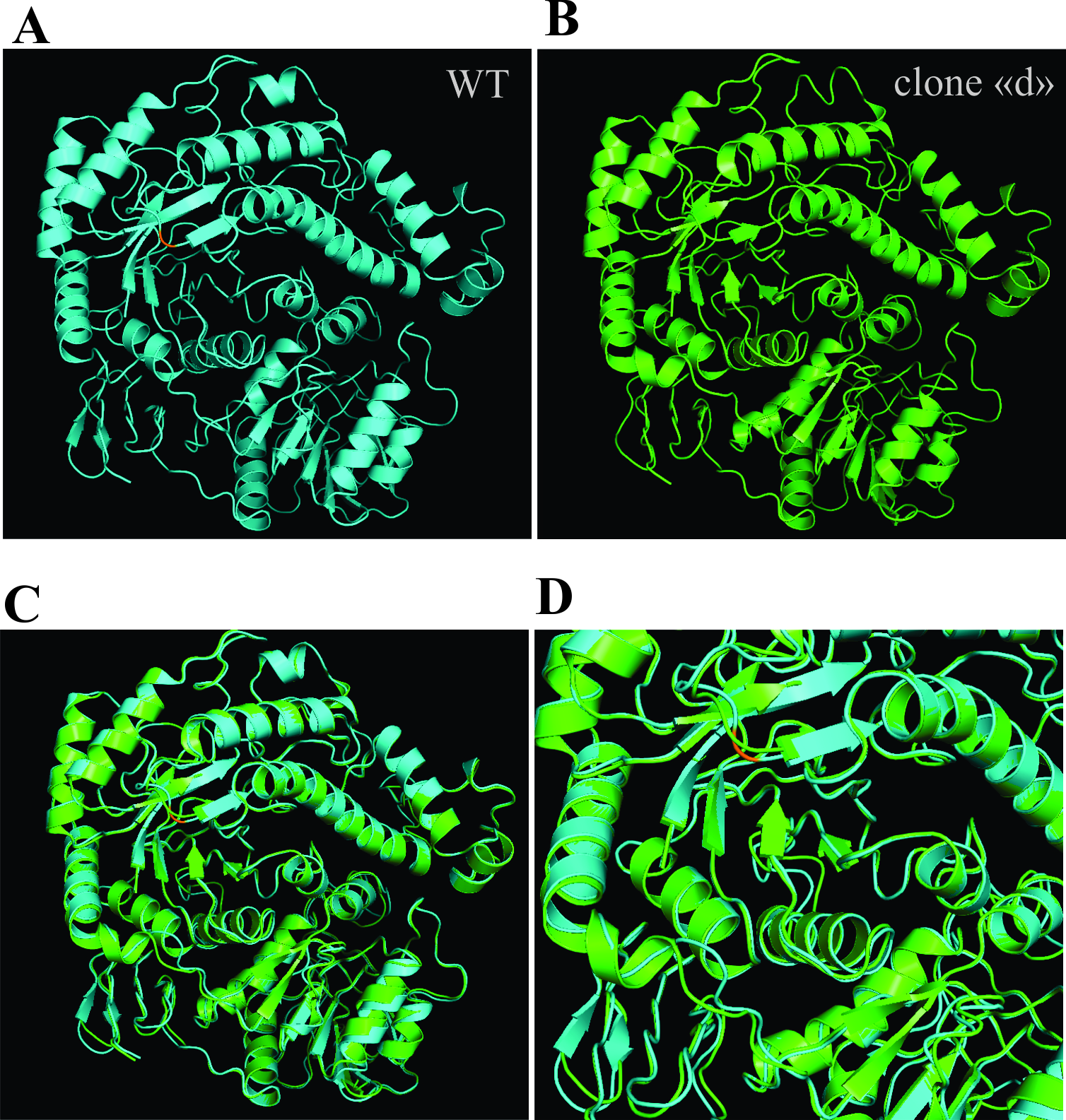

Supplement: Supplementary Figure 1 — Protein modeling of wild-type and in-frame mutants of L. biflexa β-galactosidase. Wild-type, WT (A) and mutant clone “d” (B), which had a 3-bp deletion resulting in the loss of a valine residue (shown in orange in the WT), are shown separately. Both structures were superimposed (C) and subtle changes in β-sheets could be seen close to the deletion site (D). [file Image_1.JPEG]
